# P-1398. Implementation of a Doxycycline for Post-Exposure Prophylaxis Protocol at a Large Academic HIV Clinic

**DOI:** 10.1093/ofid/ofae631.1573

**Published:** 2025-01-29

**Authors:** Rachael Pellegrino, Matthew Lokant, Nishant Patel, Christina Vojtek, Priya Jagadeesan, Andrea Ito, Richard M Merkhofer, Emily Moore, Kaitlyn Reasoner, Michael Zou, Austin Katona, Megan Turner, Jim Zhang, Christopher Terndrup, Sean Kelly, Casey Smiley

**Affiliations:** Vanderbilt University Medical Center, Nashville, Tennessee; VUMC, Nashville, Tennessee; University of Louisville School of Medicine; Vanderbilt University, Nashville, Tennessee; Vanderbilt University Medical Center, Nashville, Tennessee; Vanderbilt University, Nashville, Tennessee; Vanderbilt University Medical Center, Nashville, Tennessee; Vanderbilt University Medical Center, Nashville, Tennessee; Vanderbilt University Medical Center, Nashville, Tennessee; Vanderbilt University, Nashville, Tennessee; Vanderbilt University Medical Center, Nashville, Tennessee; Vanderbilt University Medical Center, Nashville, Tennessee; Vanderbilt University Medical Center, Nashville, Tennessee; Vanderbilt University Medical Center, Nashville, Tennessee; Vanderbilt University Medical Center, Nashville, Tennessee; Vanderbilt University Medical Center, Nashville, Tennessee

## Abstract

**Background:**

The use of doxycycline post-exposure prophylaxis (DoxyPEP) lowers the incidence of gonorrhea, chlamydia, and syphilis in men who have sex with men and transgender women living with HIV or taking pre-exposure prophylaxis (PrEP) for HIV. We aimed to implement a standardized protocol to increase the number of appropriate DoxyPEP prescriptions and decrease bacterial sexually transmitted infections (STI) in our clinic population.

**Figure:**
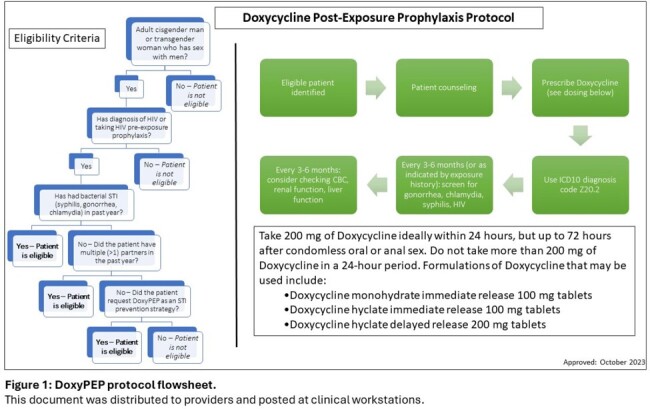

**Methods:**

Based on existing evidence, a DoxyPEP clinic protocol and flowchart (Figure 1) were developed and implemented in November 2023 at an academic clinic providing HIV and PrEP services. Interventions included provider education and display of the flowchart in clinical workspaces. The number of new DoxyPEP prescriptions was collected by chart review for the 10 months prior to and 5 months after implementation and compared by t-test. Baseline patient characteristics and STI diagnoses for the clinic population living with HIV were described. Pre- and post-implementation surveys were used to assess provider practices and comfort with DoxyPEP prescribing.

**Figure:**
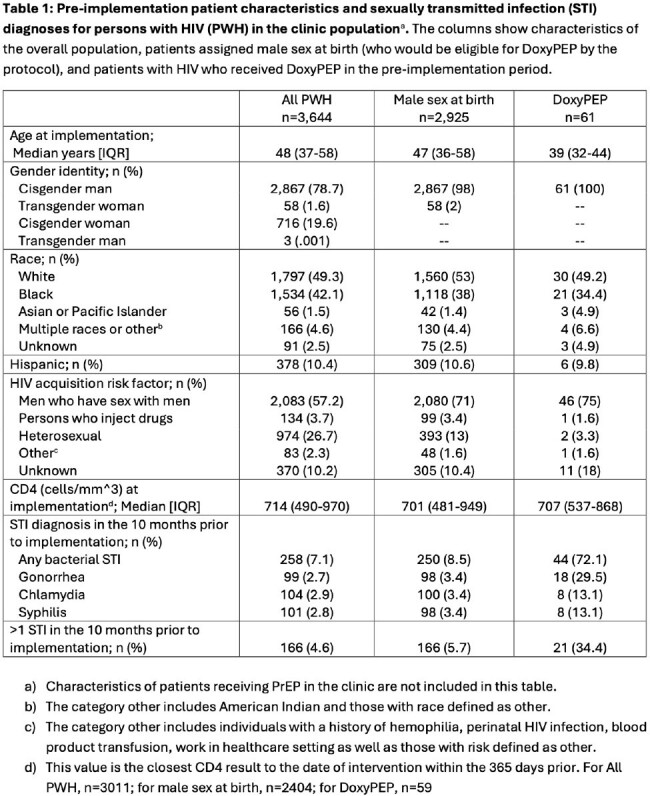

**Results:**

Of persons with HIV prescribed DoxyPEP in the pre-implementation period, 72.1% had at least 1 bacterial STI and 34.4% had >1 bacterial STI in the preceding 10 months (Table 1). There were significantly more DoxyPEP prescriptions (p< 0.001) in the 5-month post-implementation period [148 new prescriptions, 29.6/month] compared to the 10-month pre-implementation period [70 new prescriptions, 7/month] (Figure 2).

Post-implementation, there was an increase in providers who felt comfortable or very comfortable counseling a patient on DoxyPEP (86% vs 60%) and assessing a patient’s eligibility for DoxyPEP (85% vs 60%). Most respondents (86%) reported use of the protocol, and of these providers, all reported it easy or very easy to use. Nine of the fourteen DoxyPEP prescribers (64.3%) in the post-implementation period had not prescribed DoxyPEP prior to the intervention.

**Figure:**
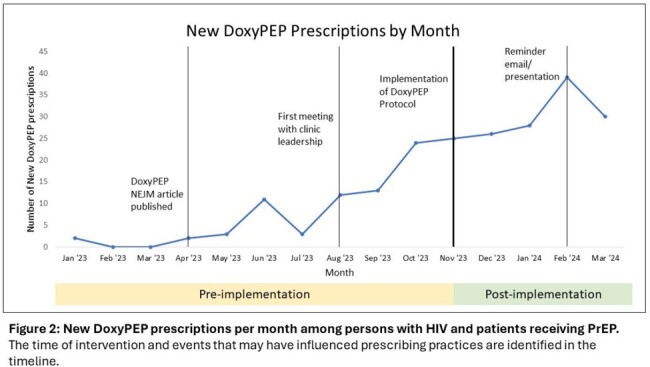

**Conclusion:**

Implementation of a standardized DoxyPEP protocol using education and updated policy increased providers’ comfort with and prescribing of DoxyPEP and can help inform future implementation efforts. Further assessment of post-implementation STI rates and patient characteristics are planned.

**Disclosures:**

**Sean Kelly, MD**, ViiV: Advisor/Consultant

